# Effect of Indian clubbell exercises on cricket fast bowlers’ shoulder kinematics

**DOI:** 10.17159/2078-516X/2023/v35i1a15103

**Published:** 2023-11-06

**Authors:** S Walter, E Moltchanova, C Petersen

**Affiliations:** 1 Faculty of Health, University of Canterbury, 20 Kirkwood Avenue, Riccarton, Christchurch 8041, New Zealand; 2 School of Mathematics and Statistics, University of Canterbury, 20 Kirkwood Avenue, Riccarton, Christchurch 8041, New Zealand

**Keywords:** rotator cuff, bowling shoulder, shoulder injury

## Abstract

**Background:**

The glenohumeral joint’s rotational range of motion (ROM) and muscle strength are essential to execute the cricket bowling action. Performing shoulder rotation exercises may increase the rotator cuff muscle strength and rotational ROM.

**Objectives:**

The aim of this study was to test the effect of a six-week exercise programme on shoulder rotational ROM and muscle strength.

**Methods:**

Twenty-one healthy male cricket fast bowlers were recruited, ranked and pair-matched on initial shoulder rotator muscle strength and assigned to either a shoulder exercise (SE) group or cricket training (CT) only group. The SE group incorporated Indian clubbell exercises in addition to their cricket training.

**Results:**

Bowlers in both groups displayed a large increase on the dominant shoulder’s internal rotation (IR) ROM, but only the SE group’s bowlers displayed ROM improvements (p<0.001) bilaterally for both internal and external rotation. The CT group’s fast bowler’s non-dominant shoulder IR ROM significantly decreased (p=0.02) during the six weeks. Between groups, only the SE group’s bowler’s internal rotator muscle strength improved (p<0.001) bilaterally. The observed kinematic changes were statistically significantly greater at a 5% level for the SE group’s bowlers (bilateral internal rotators muscle strength, non-dominant shoulder IR ROM and horizontal adduction ROM).

**Conclusion:**

Maintenance of the shoulder’s rotational ROM and muscle strength is vital for a fast bowler. Cricket bowlers who perform regular clubbell exercises might increase their shoulder’s ROM and internal rotator cuffs’ muscle strength, which may aid in stabilising their glenohumeral joint while bowling.

Bowling, fielding, and wicketkeeping are the main playing positions for the fielding team in cricket. Among these positions, the bowlers must repetitively bowl during their overs and perform throwing during fielding. One of the main differences between the spinners and the fast bowlers is the speed of the ball delivered. To bowl a quick ball, the fast bowler must repeatedly rotate the shoulder joint at faster velocities than in other playing positions.^[1]^ The shoulder muscles must produce higher torque to rotate the shoulder at faster velocities. Shoulder rotation occurs at the glenohumeral joint, and the rotator cuff muscles elicit these rotations.^[2–4]^ In addition to rotation, the rotator cuff muscles also compress the humeral head within the glenoid fossa ^[5]^ to stabilise the joint during every bowling action.^[6]^

The cricket bowling action starts with shoulder abduction, followed by external rotation (ER) and IR at the glenohumeral joint. During every bowling delivery, the shoulder abduction is initiated by the concentric contraction of the supraspinatus followed by the concentric contraction of the subscapularis and other rotator muscles creating torque to elicit IR.^[7]^ This torque is matched by the eccentric contraction of the external rotators to stabilise the humeral rotation and resist anterior humeral translation. The repetitive ER is produced by the concentric contraction of the rotator cuff and teres minor, while the subscapularis muscle's concentric contraction produces the IR.^[7]^ Therefore, to bowl multiple fast ball deliveries, glenohumeral rotation and rotator cuff muscle strength are vital.^[8]^

The volume of training and matches during a fast bowler’s career requires them to perform many explosive shoulder rotations. Short recovery periods of 20–30 seconds between deliveries, combined with the repetitive bowling action, may weaken and strain the shoulder rotators over time.^[9]^ These excessive shoulder movements may cause structural adaptations in the glenohumeral joint. These structural adaptations may present deficits in the glenohumeral rotational range of motion (ROM). Changes to the rotational ROM have been associated with shoulder pain and injury.^[10–12]^ Asymmetry between the internal and external shoulder rotators has been observed on the dominant bowling shoulders. These asymmetries might present as risk factors leading to shoulder injuries in overhead motion athletes.^[13]^ Therefore to meet the demands of fast bowling, it is recommended to strengthen the shoulder rotators and maintain the rotational ROM to minimise shoulder injury risk.^[8]^

While shoulder rotator strength and ROM improvements from exercise interventions have been observed in other overhead motion athletes ^[14],^ few studies are cricket-related. Progressive resisted exercises have shown beneficial improvement to shoulder strength among overhead-throwing athletes with shoulder pain.^[15]^ Therefore, it is essential to implement a shoulder exercise programme for cricket fast bowlers and to investigate the changes to their shoulder kinematics.

During bowling, the shoulder moves in multiple planes, involving a 180º glenohumeral circumduction.^[16]^ Using exercise equipment that could provide resistance throughout the 180º circumduction movement might be beneficial. The Indian clubbells are wooden clubbells shaped like bowling pins, with weights ranging from 1–20kg and can provide resistance while performing circumduction movements in multiple planes.^[17]^ No studies have investigated the effects of an exercise programme using Indian clubbells on the cricketer’s shoulder kinematics. This study investigated the effects of an Indian clubbell shoulder exercise programme on cricket fast bowlers’ shoulder kinematics.

## Methods

### Participants

The University of Canterbury Human Ethics Committee approved this study (HEC Application 2017/52/LR-PS). Poster advertisements invited healthy male cricket fast bowlers willing to participate in a six-week shoulder exercise programme. The inclusion criteria were no history of shoulder pain in the past 12 months, being at least 18 years of age, playing senior club cricket and having at least five years of fast bowling experience as their primary playing position. These fast bowlers were informed of the study's purpose, and their consent was obtained. Twenty-one male club cricket fast bowlers were successfully recruited. Their characteristics are presented in Table 1. The recruited bowlers actively played cricket throughout the study period and undertook two days of cricket training and one match day every week. All the fast bowlers underwent the same type, frequency, and duration of cricket training and refrained from playing any other sport during the study period. None of the fast bowlers were involved in any other form of resistance or strength training during the six weeks.

### Study design

**Fig. 1:**
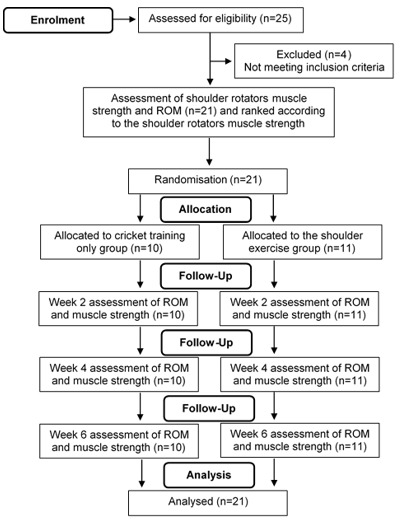
Consort flow diagram showing the flow of fast bowlers through each stage of the randomised control trial. ROM, Range of Motion.

This was a randomised control trial. The recruited fast bowlers were initially measured for their dominant arm shoulder rotator (internal rotator + external rotator) muscle strength. They were ranked based on their initial dominant arm shoulder rotator muscle strength and randomly assigned via coin toss to either a shoulder exercise group (SE) or a cricket training-only group (CT). There were no statistically significant differences in the measured variables pre-intervention between the groups. Both group bowlers underwent testing at the end of weeks two, four and six, respectively. The consort flow diagram of the study design is presented in Figure 1.

### Study setting

Participant recruitment, pre-participation assessment, delivery of the shoulder exercise programme and data collection of all the assessed variables were conducted on the cricket grounds during the team’s weekly training days.

### Testing procedures

**Fig. 2:**
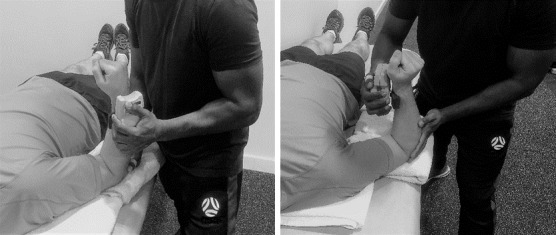
Assessment of shoulder external and internal rotators muscle strength.

All tests were measured bilaterally, with the bowlers in a supine decubitus position on an elevated plinth. A Microfet 3 handheld dynamometer (HHD) (Hoggan Scientific, LLC, Salt Lake City, UT, USA) was used to measure muscle strength. Following a warm-up consisting of ten shoulder IR and ER movements, the bowlers were familiarised with the testing procedures before the start of the testing. They were asked to perform three shoulder rotational efforts with a 30-second rest interval between each effort. Muscle strength was evaluated with the shoulder in 30º shoulder abduction and 90º elbow flexion. External rotator muscle strength was measured with the HHD positioned 2cm proximally from the styloid process of the radius on the dorsal side of the forearm. The internal rotator muscle strength was measured with the HHD positioned 2cm from the proximal wrist crease on the ventral side of the forearm. The HHD was positioned in place by the tester, and the bowlers were asked to hold their arms in position up against the rotational force for five seconds for the manual break tests, as shown in Figure 2. The instructions for the manual break tests were, “*hold as hard as you can: don’t let me move your arm*.” Consistent verbal encouragement was given during the five second contraction test period. Triplicate measures were performed with the mean used for the data analysis. Both group’s bowlers were tested the same day after their regular cricket warmup.

**Fig. 3:**
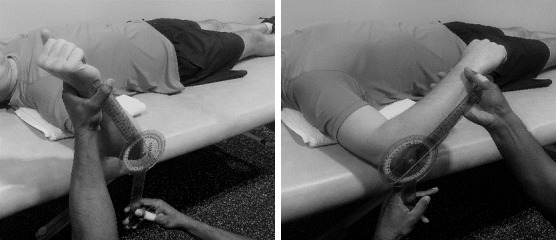
Assessment of the shoulder external and internal rotation active range of motion.

A goniometer (EGM-422-EMI 12 Elite medical instruments, Fullerton, CA, USA) was used for measuring the glenohumeral rotational ROM and shoulder horizontal adduction ROM. Shoulder IR and ER active ROM were measured, with the tested side in 90º glenohumeral abduction and 90º elbow flexion. Using the olecranon process as the axis point, the goniometer’s moving arm was moved with the forearm, while the goniometer’s fixed arm was placed perpendicular to the ground. A towel was folded and placed as a wedge between the surface of the scapula and plinth for stability, as shown in Figure 3.

**Fig. 4:**
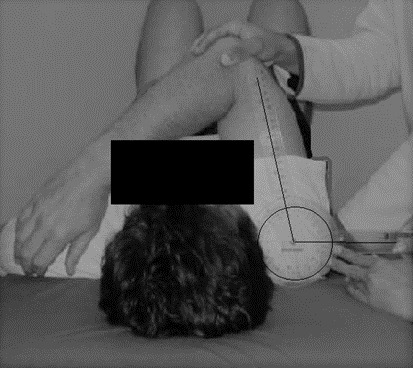
Assessment of the shoulder horizontal adduction range of motion.

Shoulder horizontal adduction ROM was measured with the shoulder at 90º abduction and elbow at 90º flexion as shown in Figure 4. The acromioclavicular joint was used as the goniometer axis, the fixed arm was perpendicular to the plinth, and the moving arm was in line with the elbow joint’s lateral epicondyle. Passive horizontal shoulder adduction was executed, and the goniometer reading was recorded. Resisted isometric muscle strength, rotational ROM and shoulder horizontal adduction ROM were measured at baseline and the end of weeks two, four and six (post-test).

### Reproducibility

The evaluator measured 11 bowlers on two separate test sessions with seven days between sessions (N=11, age 24 ± 5 years, mass 81 ± 8 kg, height 1.83 ± 0.11 m) for intra-rater reliability. The intra-class correlation coefficients (ICC) for the three trials were IR and ER ROM, ICC = 0.94 (N=11, df = 10, 95% CI: 0.84–0.98); muscle strength, ICC = 0.88 (N=11, df = 10, 95% CI: 0.71–0.96) and shoulder horizontal adduction ROM, ICC = 0.82 (N = 11, df = 10, 95% CI: 0.60–0.94).

### The shoulder exercise programme

For the duration of the exercise programme, SE fast bowlers performed Indian clubbell exercises 20 minutes per day on three non-consecutive days per week for six weeks. Indian clubbells weighing 500g each and 1kg each (Purpleheart Armoury, USA) were used. Exercise techniques were demonstrated and taught to the SE bowlers to ensure correct exercise techniques were executed. The SE bowlers were also provided with demonstration videos and exercise worksheets. Each week the SE bowlers were given three exercise worksheets that explained the execution method, frequency, intensity, and duration of each exercise. After each session, the fast bowlers were required to record the date, time, exercise sets and repetitions in their worksheets. The CT fast bowlers were not given access to the exercise programme or equipment. The Indian clubbell exercise programme is included as an appendix.

### Statistical analysis

For the sample, means and standard deviations of muscle strength and ROM were evaluated each week for each group. A repeated measures Cohen’s *d* (d^rm^) for the six-week change was also evaluated. For the muscle strength and ROM, a repeated measures ANCOVA with three factors (CT/SE group, ER/IR, and dominant/non-dominant arm) and a continuous covariate time was fitted to the data using the lmer package in R.^[18]^ A repeated measures ANCOVA with two factors (CT/SE group and dominant/non-dominant arm) and a continuous covariate time was likewise fitted for the shoulder’s horizontal adduction ROM. The repeated measures ANCOVA allows to account for repeated measurements on the same individuals and thus provides a unified, statistically powerful analysis framework. The statistical significance of the training group was tested via the chi-squared likelihood test. The individual contrasts of interest were then compared using the emmeans package. A statistical significance level of 5% was used throughout.

## Results

**Fig. 5:**
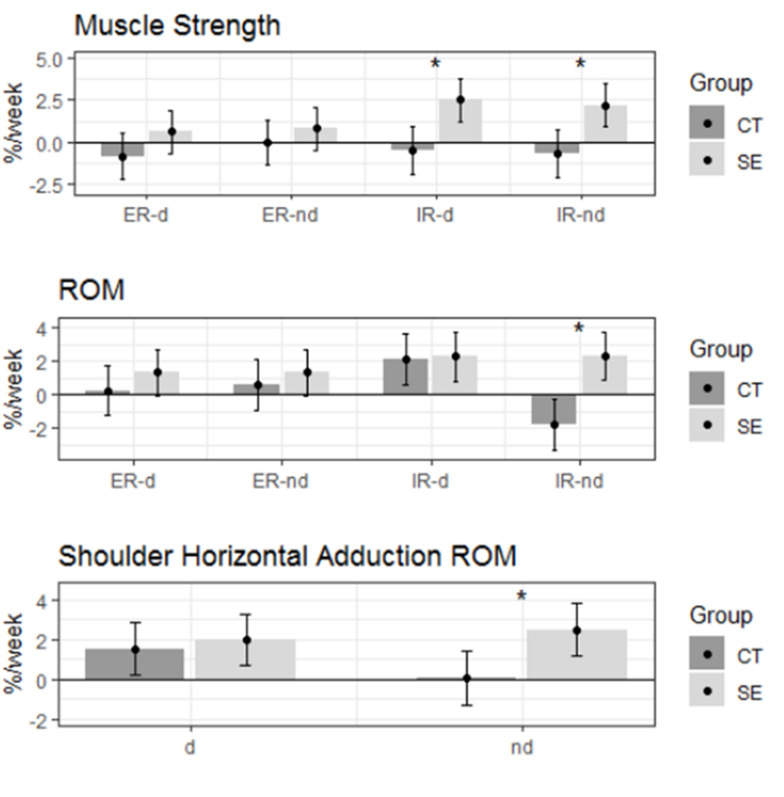
Estimated mean and 95% confidence intervals for the weekly relative change. * indicates statistically significant difference in changes between the CT and SE group bowlers (p<0.05). CT, cricket training only group; SE, shoulder exercise group; IR, internal rotation; ER, external rotation; d, dominant arm; nd, non-dominant arm; ROM, range of motion.

There were 11 bowlers in the SE group and 10 bowlers in the CT group, and their weekly sample means, standard deviations, and Cohen’s *d* for the change between the baseline week six are shown in Table 2. The estimated relative weekly change by variable and group are shown in Table 3 and Figure 5, respectively. The ANCOVA analysis showed that overall, there were statistically significant differences between the CT and SE groups for all three outcomes with χ2(8)=36.4, p<0.001 for muscle strength, χ2(8)=27.7, p<0.001 for ROM, and χ2(8)=14.9, p=0.005 for shoulder horizontal adduction ROM. The ER ROM of the non-dominant shoulder was found to increase at an average of 2.3% per week for the SE group (t(330)=3.24, p=0.001). That was statistically significantly higher than the change observed in the CT group (t(330)=3.99, p<0.001) bowlers. There was no difference in ROM changes for other conditions.

There were no statistically significant changes in muscle strength for any condition for the CT group bowlers. However, the SE group bowlers displayed a muscle strength increase of 2.5% per week and 2.2% per week on their dominant and non-dominant internal rotators, t(330)=3.85, p<0.001 and t(330)=3.36, p<0.001, respectively. Both changes were found to be statistically significantly different from the respective changes in the CT group, t(330)=3.17, p<0.001, and t(330)=3.04, p<0.001, respectively.

The shoulder horizontal adduction ROM increased for both dominant and non-dominant arms in the SE group at the rate of 2.0% and 2.5% per week, respectively. Both changes were statistically significant with t(153)=3.00, p=0.003 and t(153)=3.72, p<0.001 respectively. The change was significantly higher in the non-dominant shoulders of SE group bowlers compared to the CT group bowlers, t(153)=2.51, p=0.01. Neither group's fast bowlers complained of shoulder pain during the six weeks.

## Discussion

This study aimed to investigate the changes to shoulder kinematics after participating in a six-week Indian clubbell shoulder exercise programme. The results show a significant improvement in muscle strength and rotational and horizontal adduction ROM for the bowlers involved in the Indian clubbell exercise programme. These changes were significantly greater for the SE group bowler’s internal rotator muscle strength bilaterally and their non-dominant shoulder’s IR ROM and horizontal adduction ROM.

### Rotational range of motion changes

During the six weeks, large statistically significant improvements were observed in the IR ROM of the dominant shoulders for fast bowlers in both groups as shown in Tables 2 and 3. The observed ROM increases might be the result of the study commencing at the start of the playing season, and the bowlers adhering to regular cricket training thrice weekly. Similar changes to the rotational ROM during the playing season have been reported among baseball pitchers, but these observed changes were mainly to the dominant arm’s ER ROM.^[19]^ Baseball pitchers have been observed to display a reduction in their IR ROM during the season^[20]^, but in the current study only the CT group bowler’s non-dominant shoulders displayed a significant reduction in the IR ROM as shown in Tables 2 and 3 respectively. At the same time, because the SE group bowlers participated in the Indian clubbell exercises, their non-dominant shoulder’s IR ROM showed significantly larger improvements than the CT group, as shown in Tables 2 and 3. While comparing the between-group ROM changes, the fast bowlers in the SE group displayed moderate to large changes to their rotational ROM, which we believe is due to participation in the Indian clubbell exercise programme.

### Muscle strength changes

The internal rotator muscle’s strength significantly improved on both shoulders for the SE group fast bowlers, with an estimated 2% improvement observed during every assessment. Whereas the muscle strength of the CT group’s fast bowlers showed no strength improvements, as shown in Table 3. Handball players have been observed to decrease their internal rotator muscle strength during the season, ^[21]^ but participation in a shoulder exercise programme has been shown to improve their muscle strength.^[22]^ This indicates that in our current study, even though all fast bowlers regularly trained and played cricket, participation in the clubbell exercises might have specifically strengthened the shoulders of the SE group’s fast bowlers. The statistically significant (p=0.02) decrease observed in the shoulder rotator muscle strength of the CT group’s fast bowlers might be due to fatigue from regular cricket training. For example, softball athletes who decrease shoulder rotator strength during the season due to the onset of fatigue have been observed to develop rotator cuff tendinopathy.^[23]^ As shoulder rotator strength aids glenohumeral joint stability, regular participation in the clubbell exercise programme during the playing season will strengthen the glenohumeral internal rotators.

With no change observed in the strength of the external rotators in the current study, it is important to focus on the external rotators to try and limit the imbalances that are already caused by excessive use of the internal rotators. Therefore it is suggested that the exercises employed require modification to target the external rotators. Even though the training type, frequency and duration of cricket played by both groups was the same, the addition of the clubbell exercise programme did not cause any injury or discomfort to the SE fast bowlers.

### Shoulder horizontal adduction changes

Detecting ROM deficits is critical for predicting injury risk, and evidence suggests that ROM deficits can be rectified.^[24]^ Glenohumeral joint internal rotation deficit is associated with shoulder horizontal adduction ROM deficit, which has been commonly reported amongst overhead motion athletes.^[25]^ Therefore we wanted to find out if there were any changes to the shoulder horizontal adduction ROM during the six weeks of the programme. The results reveal that significant improvements were observed in the fast bowlers' dominant shoulders. But on the non-dominant side, only the SE group fast bowlers displayed a large improvement. The ROM changes observed in the dominant side of both groups may be due to cricket participation, but the changes observed in the non-dominant side of the SE group bowlers could be mainly due to participation in the clubbell exercise programme. The changes to shoulder horizontal adduction ROM were monitored during the study period, and as these fast bowlers were healthy cricketers, the observed IR ROM deficit and shoulder horizontal adduction ROM values were not critical. If these same measurements were undertaken on injured players, values would likely be lower.

As both group’s bowlers were actively playing and training for cricket, it is difficult to determine how much of the strength and ROM adaptations were solely due to the exercise programme. Future research with a crossover design incorporating a washout period is recommended. Conducting the same study with a larger sample of fast bowlers training in the same squad with the same training frequency and duration is also necessary to establish the statistical power.

## Conclusion

This study investigated the effectiveness of an Indian clubbell shoulder exercise programme on cricket fast bowlers' shoulder kinematics. Based on the findings of the study, we suggest that Indian clubbell exercises may strengthen the shoulder’s internal rotators, increase shoulder rotational ROM, and increase shoulder horizontal adduction ROM.

## Supplementary Information



## Figures and Tables

**Table 1:** Participant characteristics

Group	Mass (kg)	Height (m)	Age (y)	Arm dominance right/left	Cricket playing experience (y)
CT (n= 10)	81 ± 8	1.8 ± 0.1	24 ± 4	7/3	6 ± 1
SE (n= 11)	83 ± 8	1.8 ± 0.5	24 ± 6	9/2	7 ± 2

Data are presented as mean ± SD where appropriate. CT, cricket training only group; SE, shoulder exercise group; y, years

**Table 2:** Observed range of motion and muscle strength throughout the six weeks

Variable	Group	Arm	Baseline	Week 2	Week 4	Week 6	Cohen’s d
**Range of motion (°)**	**CT**	ER dominant	74 ± 8	72 ± 6	73 ± 11	75 ± 4	0.10
		IR dominant	61 ± 2	62 ± 2	67 ± 8	68 ± 6	1.40
		ER non-dominant	71 ± 8	68 ± 5	71 ± 7	73 ± 5	0.22
		IR non-dominant	68 ± 6	60 ± 2	61 ± 10	61 ± 6	0.81

	**SE**	ER dominant	74 ± 9	78 ± 9	79 ± 11	80 ± 6	0.70
		IR dominant	58 ± 6	65 ± 7	65 ± 7	67 ± 6	1.32
		ER non-dominant	71 ± 9	72 ± 9	74 ± 11	77 ± 5	0.57
		IR non-dominant	64 ± 10	64 ± 11	73 ± 6	70 ± 6	0.76

**Muscle strength (Nm)**	**CT**	ER dominant	39 ± 5	39 ± 6	38 ± 5	38 ± 4	0.41
		IR dominant	43 ± 7	43 ± 6	41 ± 8	42 ± 6	0.13
		ER non-dominant	38 ± 8	37 ± 6	37 ± 6	37 ± 5	0.09
		IR non-dominant	42 ± 7	41 ± 7	40 ± 8	40 ± 6	0.25

	**SE**	ER dominant	42 ± 5	38 ± 6	41 ± 7	42 ± 6	0.11
		IR dominant	39 ± 7	41 ± 5	44 ± 6	45 ± 6	0.97
		ER non-dominant	41 ± 4	40 ± 6	42 ± 5	42 ± 5	0.37
		IR non-dominant	39 ± 5	40 ± 8	42 ± 6	45 ± 6	1.00

**Shoulder horizontal adduction ROM (°)**	**CT**	Dominant	32 ± 5	34 ± 5	35 ± 4	35 ± 3	0.78
		Non-dominant	36 ± 3	37 ± 4	36 ± 4	36 ± 4	0.07

	**SE**	Dominant	33 ± 4	36 ± 3	37 ± 5	37 ± 4	1.04
		Non-dominant	31 ± 4	35 ± 4	35 ± 5	37 ± 3	1.52

Data are presented as mean ± SD. CT, cricket training only group; SE, shoulder exercise group; IR, Internal rotation; ER, External rotation; Nm, Newton metre; ROM, Range of Motion. Cohen’s d effect sizes are typically interpreted as trivial if d ≤ 0.2, small if d = 0.2 – 0.6, moderate if d = 0.6 – 1.2 and large if d = 1.2 – 2.0.

**Table 3:** Estimated relative weekly changes (% per week)

Variable	Group	Arm	Estimate	95% Cl	p-value
**Change in range of motion (%)**	**CT**	ER dominant	0.2	(−1.2, 1.7)	0.74
		IR dominant	2.1	(0.6, 3.6)	0.006*
		ER non-dominant	0.6	(−0.9, 2.1)	0.44
		IR non-dominant	−1.8	(−3.3, −0.3)	0.02*

	**SE**	ER dominant	1.3	(−0.1,2.7)	0.08
		IR dominant	2.3	(0.8, 3.7)	0.002*
		ER non-dominant	1.3	(−0.1, 2.7)	0.07
		IR non-dominant	2.3	(0.9, 3.7)	0.001*

**Change in muscle strength (%)**	**CT**	ER dominant	−0.9	(−2.2, 0.5)	0.21
		IR dominant	−0.5	(−1.9, 0.9)	0.48
		ER non-dominant	0.0	(−1.4, 1.3)	0.96
		IR non-dominant	−0.7	(−2.1, 0.7)	0.32

	**SE**	ER dominant	0.6	(−0.7,1.9)	0.40
		IR dominant	2.5	(1.2, 3.8)	<0.001*
		ER non-dominant	0.8	(−0.5, 2.1)	0.21
		IR non-dominant	2.2	(0.9, 3.5)	<0.001*

**Change in shoulder horizontal adduction ROM (%)**	**CT**	Dominant	1.5	(0.2, 2.9)	0.03*
		Non-dominant	0.1	(−1.3, 1.4)	0.94

	**SE**	Dominant	2.0	(0.7, 3.3)	0.003*
		Non-dominant	2.5	(1.2, 3.8)	<0.001*

* indicates statistical significance (p<0.05).

CT, cricket training only group; SE, shoulder exercise group; IR, Internal rotation; ER, External rotation; ROM, Range of Motion.
