# Supplementary material for: Effect of Indian clubbell exercises on cricket fast bowlers’ shoulder kinematics
Source: S Afr J Sports Med. 2023 Nov 6;35(1):v35i1a15103. doi: 10.17159/2078-516X/2023/v35i1a15103 (PMC10798617; doi:10.17159/2078-516X/2023/v35i1a15103)
Supplement: Supplementary file 1 [file 2078-516X-35-v35i1a15103-s001.pdf]

Indian Clubbell Shoulder Exercise Programme

Execute all the exercises at a slow controlled pace maintaining a rhythmic movement.

Exercise	Procedure	Image	Sets	Reps
90^0 Abduction Upright Rotations	Hold the clubbells at 90^0 shoulder abduction and rotate it internally and externally	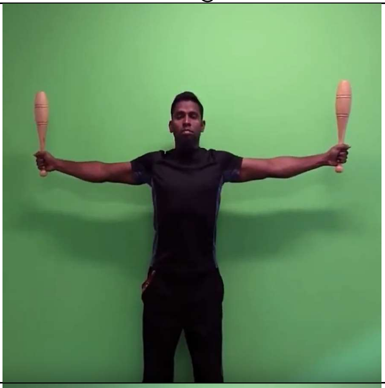	3	10
90^0 Abduction Upside Down Rotations	Hold the clubbells upside down at 90^0 shoulder abduction and rotate it internally and externally	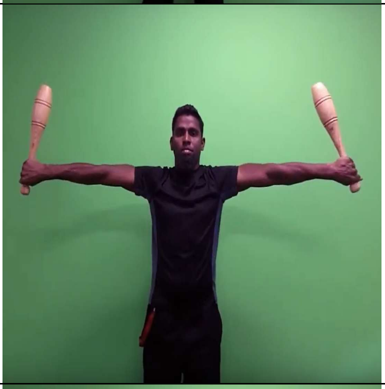	3	10
Scapular Plane Upright Rotations	Hold the clubbells in the scapular plane upright and rotate it internally and externally	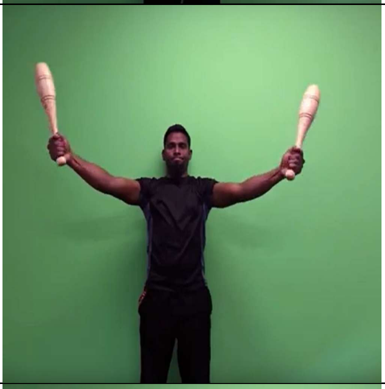	3	10
Scapular Plane Upside Down Rotations	Hold the clubbells in the scapular plane upside down and rotate it internally and externally	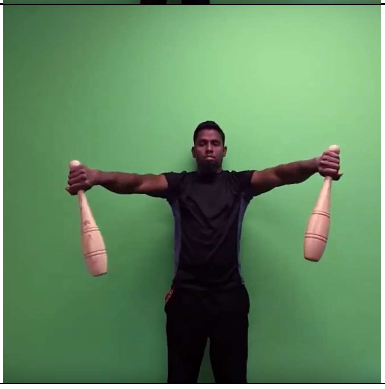	3	10

90^0 Sagittal Axis Rotations	Hold the clubbells at 90^0 shoulder flexion and perform shoulder rotations	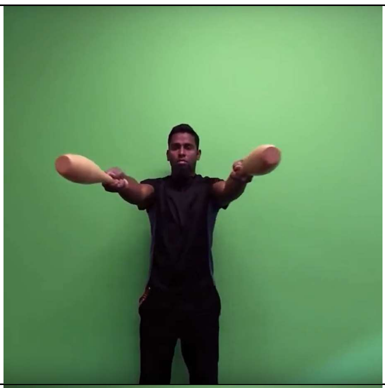	3	15
90^0 Frontal Axis Rotations	Hold the clubbells at 90^0 shoulder abduction and perform shoulder rotations	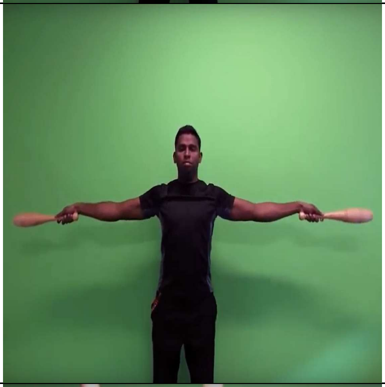	3	15
90^0 Vertical Axis Rotations	Hold the clubbells at 180^0 shoulder flexion and perform shoulder rotations	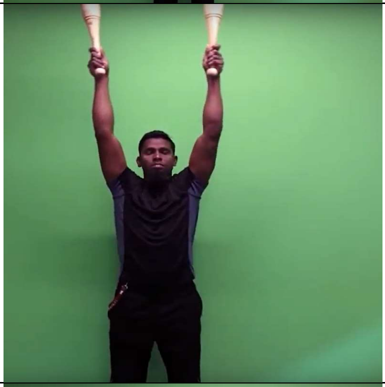	3	15
90^0 Scapular Retractions	Hold the clubbells at 90^0 flexion, and perform horizontal abduction and finish the movement with maximum scapular retraction	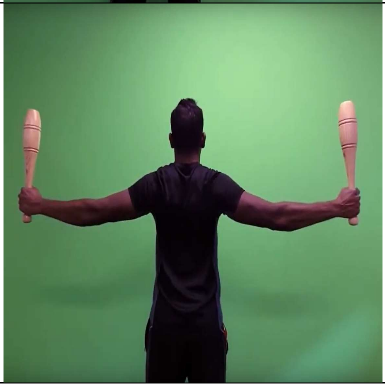	3	15

Shoulder Abduction & Internal Rotation	Hold the clubbells at 90^0 abduction, and perform horizontal adduction and finish the movement with one forearm covering the face and another forearm behind the head	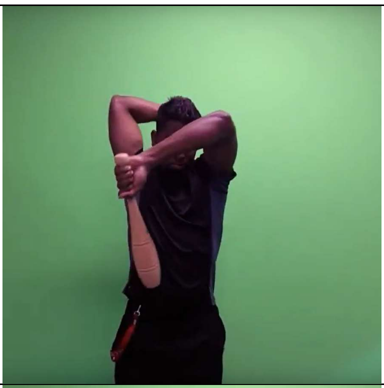	3	15
Shoulder Abduction & Adduction	Hold the clubbells at 90^0 abduction, and perform horizontal adduction and go around the head finish the movement in 90^0 abduction	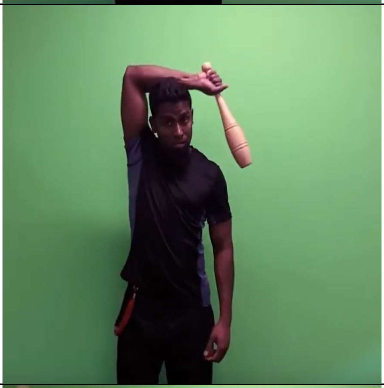	3	15
Standing Bowling Action	Standing in a split stance hold the clubbells in both hand and perform a controlled slow pace bowling action	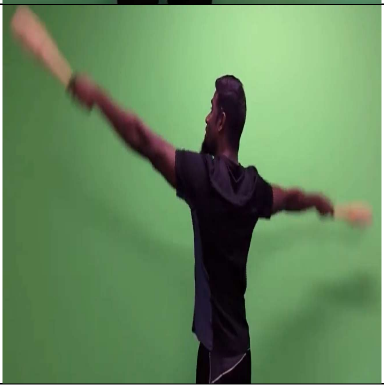	3	6
